# Unified protein–small molecule graph neural networks for binding site prediction

**DOI:** 10.1073/pnas.2524913123

**Published:** 2026-03-03

**Authors:** Jian Wang, Nikolay V. Dokholyan

**Affiliations:** ^a^Department of Neurology and Neuroscience, University of Virginia, School of Medicine, Charlottesville, VA 22903; ^b^Department of Neurology, University of Virginia, School of Medicine, Charlottesville, VA 22903; ^c^Departments of Neuroscience, Biomedical Engineering, Pharmacology, Microbiology, Immunology, & Cancer Biology, University of Virginia, School of Medicine, Charlottesville, VA 22903

**Keywords:** protein pocket prediction, protein binding site, druggable site identification

## Abstract

Predicting small molecule binding sites on proteins remains a key challenge in structure-based drug discovery. While AlphaFold3 has transformed protein structure prediction, accurate identification of functional sites such as ligand binding pockets remains a distinct and unresolved problem. Graph neural networks have emerged as promising tools for this task, but most current approaches focus on local structural features and are trained on relatively small datasets, limiting their ability to model long-range protein–ligand interactions. Here, we develop YuelPocket, a graph neural network that addresses these limitations. YuelPocket operates in two complementary modes: residue-level prediction for identifying contact residues and coordinate-level prediction for pinpointing pocket centers. Trained on the large-scale PLINDER dataset, YuelPocket achieves higher success rates in both Distance to Closest Atom and Center-to-Center metrics compared to the state-of-the-art methods. Crucially, YuelPocket demonstrates high robustness on AlphaFold-predicted structures, maintaining high accuracy for targets with deviations from experimental structures. We hope that YuelPocket will serve as a robust framework for accurate binding site identification, enabling reliable functional annotation and structure-guided drug discovery.

## Significance

Accurately identifying small molecule binding sites on proteins is fundamental to understanding protein function and enabling structure-based drug discovery, yet this critical step remains a major bottleneck in biomedical research and therapeutic development. Failures in virtual screening and lead optimization are often attributable to incorrect binding site identification rather than limitations in docking algorithms or scoring functions. We present YuelPocket, a unified graph neural network that overcomes this fundamental challenge by integrating both local and global protein–small molecule interactions within a single, scalable framework. YuelPocket achieves high predictive accuracy and offers a robust solution for precise binding site detection, providing a transformative tool for improving virtual screening and rational drug design.

Small molecule binding site prediction (1) on proteins represents one of the most critical challenges in computational biology and drug discovery, with far-reaching implications for understanding protein function, rational drug design (2, 3), and structure-based drug discovery pipelines (4–7). The accurate identification of binding pockets is fundamental to virtually every aspect of modern drug development, from target validation (8) to lead optimization and clinical candidate selection.

The pharmaceutical industry faces unprecedented challenges in drug discovery, with development costs exceeding $2.6 billion per approved drug and failure rates nearing 90% in clinical trials (9). A major factor contributing to these failures is the difficulty in accurately predicting protein–small molecule interactions. Traditional drug discovery methods often depend heavily on experimental techniques, which are costly and time-consuming. The introduction of AlphaFold2 (10) has transformed protein structure prediction, achieving unmatched accuracy in determining three-dimensional protein structures from amino acid sequences. The release of AlphaFold3 (11) has further enabled researchers to directly predict protein–small molecule complexes, potentially significantly speeding up drug discovery. However, despite its impressive capabilities, AlphaFold3 still encounters notable challenges in drug discovery. Shen et al. (12) found that while AlphaFold3 reliably predicted the overall structure of receptors, its precision in positioning small molecule ligands was variable and frequently inaccurate. This inconsistency was especially evident with allosteric modulators, highlighting a key obstacle in reliably identifying binding sites.

Existing pocket prediction algorithms can be broadly categorized into three main approaches, each with significant limitations. Traditional geometric approaches such as Fpocket (1), SiteHound (13), and CASTp (14) rely on geometric descriptors including surface curvature, solvent accessibility, and cavity detection algorithms. These methods identify potential binding sites based on physical properties such as surface concavity, pocket depth, solvent accessibility, and geometric complementarity to spherical probes. While these methods offer fast computation and interpretable results without requiring training data, they suffer from limited accuracy and poor performance on flat binding sites or allosteric sites.

More recent machine learning approaches employ various techniques to improve binding site prediction accuracy. P2Rank (15) uses a Random Forest classifier with geometric and evolutionary features to predict ligand binding sites on a protein’s solvent-accessible surface, trained on the CHEN11 (16) dataset containing 251 proteins with 476 ligands. DeepSite (17) utilizes 3D convolutional neural networks that process 16 Å^3^ protein subgrids with eight feature channels, trained on the scPDB v.2013 database with 7,622 binding sites. DeepPocket (18) employs 3D Convolutional Neural Networks for binding site detection and segmentation, trained on the scPDB (19) v.2017 database with 17,594 binding sites from 16,612 proteins. PUResNet (20) combines U-Net (21) and ResNet (22) architectures to predict binding site probabilities for each voxel in 3D protein structures. While these methods offer improved accuracy over geometric approaches, they are limited by computational intensity and difficulty in capturing long-range interactions.

Emerging graph neural network (GNN) (23)-based approaches provide more sophisticated modeling of protein structures, but suffer from fundamental limitations in both architecture and training data scale. PocketMiner (24) uses a geometric vector perceptron graph neural network to predict cryptic pockets, though it was trained on only 37 proteins from molecular dynamics simulations, severely limiting its generalization capabilities. SiteRadar (25) represents protein structures as graphs where nodes are heavy atoms and edges are interatomic distances, predicting binding sites on a grid by classifying each grid point as pocket or nonpocket. LigBind (26) employs a relation-aware graph neural network to predict ligand-specific binding residues, representing each residue and its surrounding structural context (within 15 Å radius) as a graph, with a two-phase transfer learning approach trained on over 1,000 ligands. While these methods represent advances over traditional approaches, they share critical architectural limitations: They construct only local graphs that capture limited spatial relationships between neighboring elements, fundamentally missing the global interaction patterns and synergistic binding effects where distant residues cooperatively contribute to ligand recognition. Particularly, SiteRadar creates graphs between grid points and their surrounding protein atoms, while LigBind builds graphs between residues and their neighboring protein residues within a fixed radius. Furthermore, these methods do not explicitly incorporate ligand information during the prediction process, limiting their ability to predict ligand-specific binding sites, and their training datasets remain relatively small compared to the diversity of protein–small molecule interactions in nature.

**Fig. 1.**
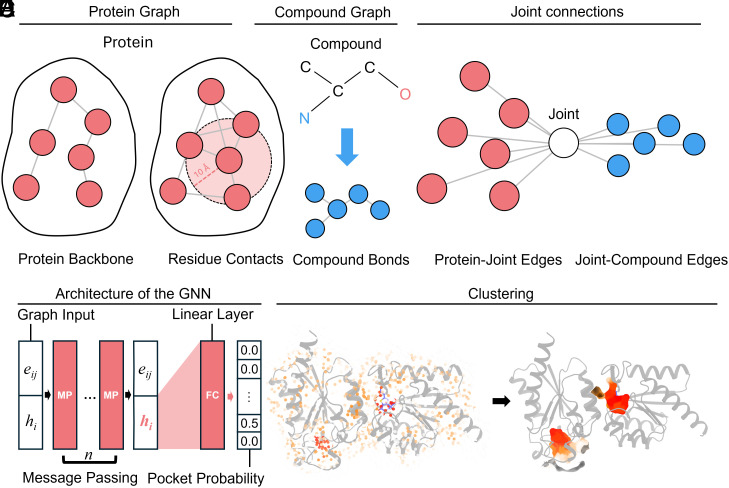
Structural and topological representation of protein–compound interactions in YuelPocket’s graph neural network framework. (*A*) Protein graph: Red nodes depict Cα atoms (spheres) with edges (black lines) representing backbone connectivity (labeled “Backbone”) and interresidue contacts (“Contacts”). (*B*) Compound graph: Blue nodes show heavy atoms (C, N labeled) with bond edges (“Bonds”); stick model overlay illustrates chemical structure. (*C*) Joint connection topology: Virtual joint node (labeled “Joint”) mediates protein–compound interactions via two edge types: “Protein-Joint Edges” and “Joint-Compound Edges”, avoiding all-to-all residue–atom connections. (*D*) GNN architecture: Graph input (*Left*) passes through message-passing layers (center) with learned edge weight updates, culminating in pocket probability predictions (*Right*). (*E*) Spatial clustering: Euclidean embedding of protein residues (colored by cluster ID) reveals pocket localization patterns.

Here, we develop an innovative graph neural network architecture, YuelPocket, to address these fundamental limitations through a global protein–small molecule interaction graph (Fig. 1). Unlike existing GNN approaches that rely on local graph construction, we introduce a virtual joint node (Fig. 1*C*) that serves as a global information hub, connecting all protein residues and all ligand atoms in a unified computational framework. The virtual joint node acts as a computational bridge that aggregates information from all protein residues and ligand atoms, and distributes the processed information back to the protein and the compounds. This design enables the linear computational complexity *O(C + m + n)* instead of the quadratic complexity *O(C + m × n)* that would result from direct all-to-all connections between protein residues and compound atoms, where *C* represents the summation of the number of protein backbones (Fig. 1*A*), residue contacts (Fig. 1*A*), and compound bonds (Fig. 1*B*), *m* represents the number of protein residues, and *n* represents the number of ligand atoms.

YuelPocket operates in a residue-level prediction mode and a coordinate-level prediction mode. The residue-level prediction mode identifies specific protein residues involved in binding, providing granular contact information essential for understanding interaction mechanisms. The coordinate-level prediction mode directly predicts the 3D coordinates of binding pocket centers by ranking Solvent Accessible Surface (SAS) probes, offering precise starting points for downstream tasks like molecular docking. We evaluated YuelPocket on the PLINDER (27) and Holo4k benchmarks, demonstrating higher performance over state-of-the-art methods. Crucially, recognizing the paradigm shift in structural biology, we assessed the robustness of YuelPocket on AlphaFold (AF)-predicted structures. We further demonstrate its utility through a minimal probe set approach, enabling the comprehensive exploration of binding space across diverse targets.

## Results

### Residue-Level Evaluation on Benchmark Datasets.

We utilized the PLINDER dataset (v2024-06), a high-quality and comprehensive resource of protein–ligand complexes, for model training and evaluation. To minimize information leakage and enable a rigorous assessment of generalization, PLINDER adopts a similarity-based clustering strategy for its data splits. Systems are partitioned so that the training, validation, and test sets are separated by thresholds of 30% for protein sequence similarity, 30% for protein–ligand interaction (PLI) similarity, and 50% for ligand Tanimoto similarity. Under this scheme, the dataset includes 309,140 training systems, 832 validation systems, and a curated test split of 1,036 complexes. The test set is selected to ensure high-quality experimental structures and diversity in pocket types. Our model was trained on the full training split and evaluated on this 1,036-complex test set to measure residue-level prediction performance.

Holo4k is a widely used benchmark dataset for residue-level pocket prediction. To ensure a rigorous evaluation, we filtered the original 4,543 systems in Holo4k to remove overlap with PLINDER training and validation data, resulting in a set of 340 complexes. Of these, 40 overlap with the PLINDER test split (for cross-validation consistency), while 300 represent completely novel protein pockets absent from PLINDER. This selection enables testing of the model on both partially familiar and entirely new complexes, providing insight into generalization to unseen pockets.

**Fig. 2.**
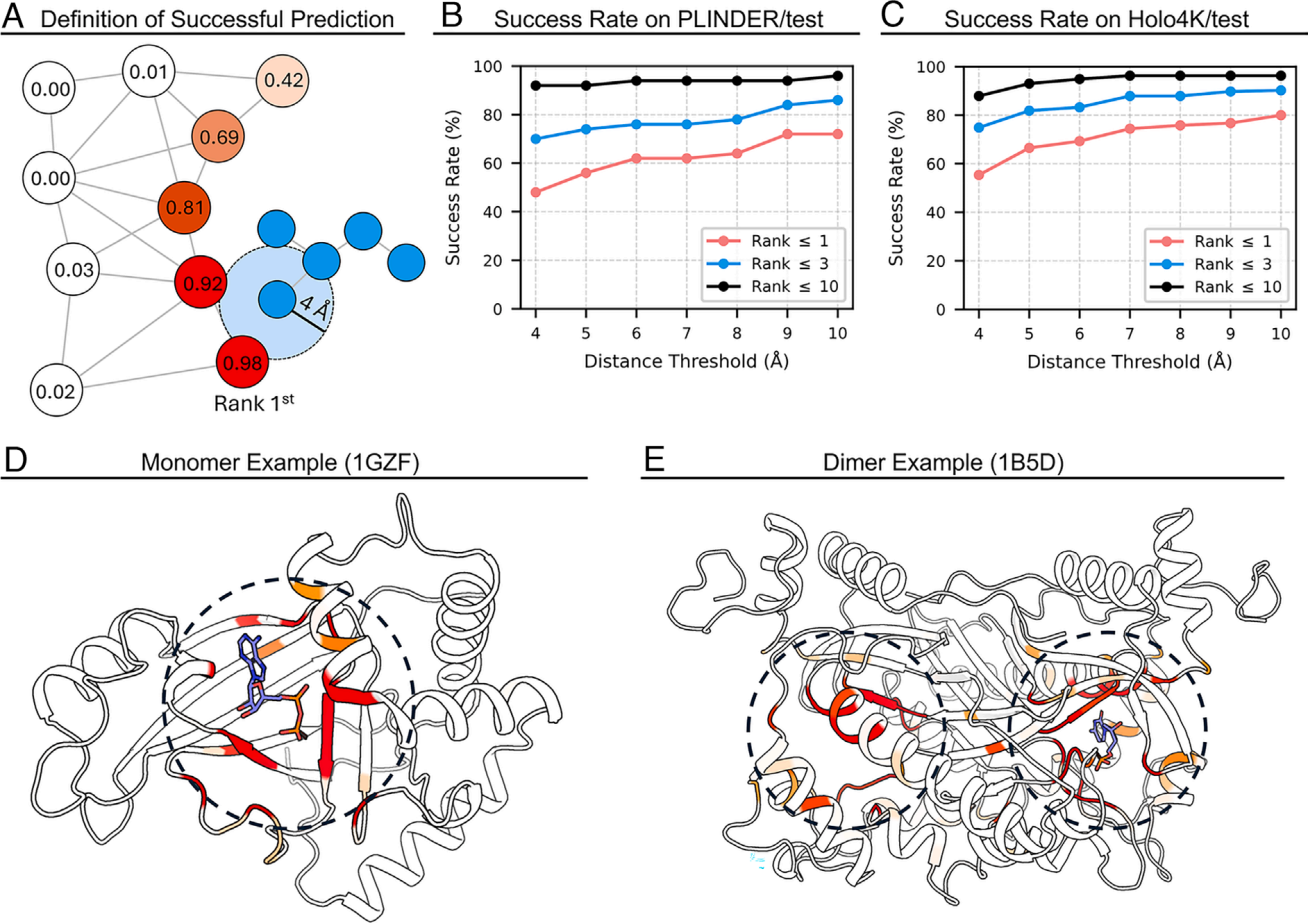
Residue-level performance of YuelPocket on benchmark datasets. (*A*) Schematic definition of a successful prediction. A prediction is considered successful if the top-ranked residue falls within a defined distance (e.g., 4 Å) of any ligand atom. (*B*) Success rates on the PLINDER test set (1,036 systems) at varying distance thresholds (4 to 10 Å) for Top-1, Top-3, and Top-10 ranked residues. (*C*) Success rates on the Holo4K test set (340 systems) under the same evaluation criteria, demonstrating robust generalization. (*D*) Visualization of predicted binding probabilities for a monomeric protein (PDB: 1GZF). Residues are colored by probability (red: high, white: low), showing accurate pocket localization. (*E*) Visualization for a dimeric protein complex (PDB: 1B5D), highlighting the model’s ability to identify binding sites in multimeric structures.

Residue-level success was assessed based on spatial distance between predicted residues and ligand atoms (Fig. 2*A*). A prediction was considered successful if the Top-N ranked residues (Top-1, Top-3, Top-10) fell within a specified distance threshold (4 to 10 Å) of any ligand atom. Using multiple Top-N levels and a range of distance thresholds provides a more nuanced evaluation, capturing both the accuracy of the highest-ranked residues and the broader coverage of potential binding sites.

On the PLINDER test set (Fig. 2*B*), at the strict 4 Å threshold, the Top-1 success rate is ~48%, increasing to over 70% for Top-3 and exceeding 90% for Top-10. These trends illustrate how increasing the number of considered residues or relaxing the distance criterion affects the likelihood of including residues that are proximal to the ligand. As the distance threshold increases to 10 Å, the Top-1 success rate rises to ~70%, reflecting that some residues slightly outside the strict 4 Å cutoff are still physically relevant to ligand binding.

Evaluation on the Holo4k dataset (Fig. 2*C*) follows the same criteria, with a Top-1 success rate of ~55% at 4 Å, and Top-3 and Top-10 success rates reaching ~75% and ~88%, respectively. Comparing the results across these two datasets allows us to examine how the model performs on datasets with different levels of complexity and composition. Notably, performance on Holo4k is slightly higher than on PLINDER, suggesting that Holo4k may include complexes that are, on average, less challenging for residue-level prediction under the chosen thresholds. By reporting results across multiple Top-N levels and distance cutoffs on two datasets, we aim to provide a comprehensive picture of residue prediction behavior, capturing both the accuracy of the highest-ranked residues (e.g., Top-1) and the broader coverage of potential binding sites (e.g., Top-10) at different spatial thresholds, rather than relying on a single metric such as Top-1 success rate at 4 Å.

### Coordinate-Level Prediction Performance and Comparison with State of the Art.

To evaluate coordinate-level prediction performance, we compared YuelPocket with P2Rank, a widely used template-free pocket prediction tool. Evaluations were performed on both the PLINDER test split and the Holo4k dataset. Two standard metrics were used: Distance to Closest Atom (DCA) and Distance Center-to-Center (DCC). DCA measures the shortest distance from the predicted pocket center to any ligand atom, providing a direct estimate of how well the predicted site overlaps with the ligand. DCC calculates the distance between the predicted pocket center and the centroid of the ligand, reflecting how accurately the overall geometry of the binding site is captured. Using both metrics allows a more complete understanding of coordinate-level performance, as each emphasizes a different aspect of spatial alignment. A prediction was considered successful if the distance between the predicted center and the ligand fell within a specified cutoff. Multiple cutoff thresholds were tested to examine sensitivity to the strictness of spatial criteria.

**Fig. 3.**
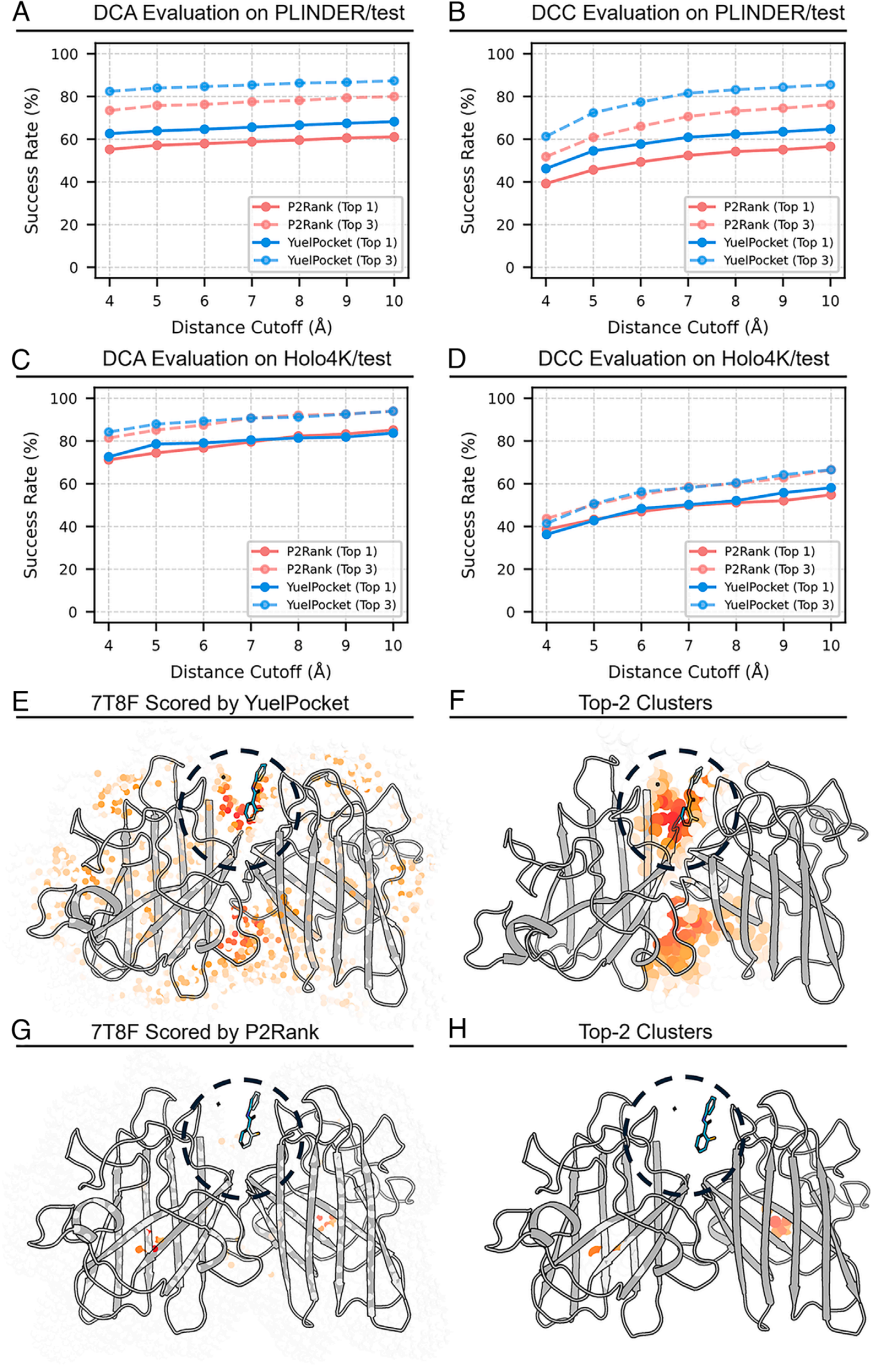
Coordinate-level performance comparison between YuelPocket and P2Rank. (*A* and *B*) Success rates on the PLINDER test set using (*A*) DCA and (*B*) DCC metrics. YuelPocket (blue) consistently outperforms P2Rank (red) in both Top-1 (solid) and Top-3 (dashed) ranks. (*C* and *D*) Success rates on the Holo4K test set for (*C*) DCA and (*D*) DCC metrics, showing similar superior performance for YuelPocket. (*E*–*H*) Visual comparison on protein 7T8F. (*E*) Raw SAS probe scores from YuelPocket and (*F*) resulting top 2 clusters. (*G*) P2Rank predictions and (*H*) resulting clusters. YuelPocket provides a clearer and more accurate definition of the binding pocket.

On the PLINDER test set, the Top-1 DCA success rate for YuelPocket is ~62% at a 4 Å cutoff, compared with ~55% for P2Rank (Fig. 3*A*). The DCC metric shows similar trends (Fig. 3*B*), with Top-1 success rates of ~45% and ~40%, respectively. On the Holo4k dataset, performance of the two methods is more comparable (Fig. 3 *C* and *D*).

Qualitative comparison on protein 7T8F (Fig. 3 *E*–*H*) further illustrates these differences. YuelPocket generates a dense, high-probability point cloud (Fig. 3*E*) that, after clustering (Fig. 3*F*), accurately identifies the binding site with the primary cluster. In contrast, P2Rank predictions for this system are sparser (Fig. 3 *G* and *H*) with lower definition and confidence, and the clustered pocket center deviates from the GT.

### Evaluation of Robustness on AlphaFold Models.

Robust pocket prediction requires reliable performance not only on high-quality crystal structures but also on predicted protein models, which are increasingly used in modern drug discovery pipelines. To assess this aspect, AlphaFold was used to predict the structures of all 1,036 proteins in the PLINDER test split, and YuelPocket was evaluated on these predicted structures. This setup allows examination of how the model performs when applied to computationally derived protein structures rather than experimentally determined ones.

**Fig. 4.**
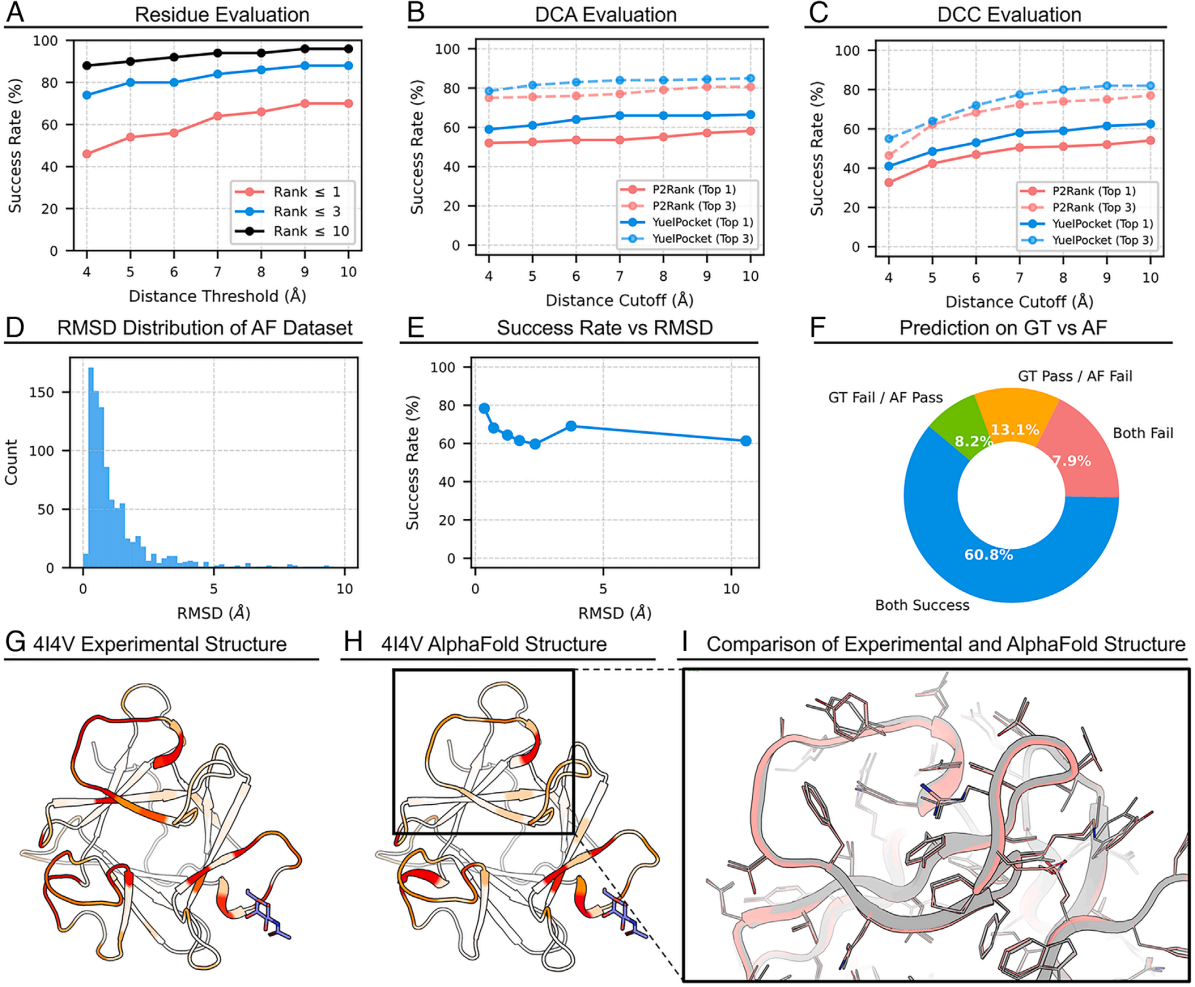
Robustness of YuelPocket on AlphaFold-predicted structures. (*A*) Residue-level success rates on the AlphaFold dataset show high accuracy (Top-10 > 90%). (*B* and *C*) Success rates on the AlphaFold dataset using DCA (*B*) and DCC (*C*) metrics. YuelPocket (blue) consistently achieves higher success rates than P2Rank (red). (*D*) Distribution of Cα RMSD between AlphaFold models and experimental crystal structures. (*E*) P2Rank and YuelPocket success rates stabilize after an initial drop as RMSD increases, demonstrating robustness. (*F*) Consistency analysis showing high overlap (blue) between successful predictions on GT and AF structures. (*G*–*I*) Visual comparison of protein 4I4V. The model correctly identifies the binding site on both the Experimental structure (*G*) and the AlphaFold model (*H*), despite sidechain deviations visible in the superposition (*I*).

At the residue level (Fig. 4*A*), YuelPocket maintains performance comparable to that on experimental PLINDER structures, achieving a Top-3 success rate exceeding 70% at a 4 Å distance threshold. When benchmarked against P2Rank using coordinate-based metrics (DCA and DCC), YuelPocket consistently outperforms P2Rank (Fig. 4 *B* and *C*); for example, the Top-1 DCA success rate is approximately 10 percentage points higher, highlighting YuelPocket’s ability to accurately identify binding sites even on predicted protein structures.

To examine the effect of structural quality, RMSD distributions of the AlphaFold models were analyzed (Fig. 4*D*). Most AlphaFold models are highly accurate (<2 Å RMSD), but a subset exhibits larger deviations. The residue-level Top-3 success rate at 4 Å decreases slightly as RMSD increases from 0 to 4 Å and then plateaus (Fig. 4*E*). At lower RMSD values, deviations primarily reflect inaccuracies in side-chain positioning rather than backbone conformation. As RMSD increases, side-chain misplacement contributes to the decline in success rate, showing that YuelPocket, despite using a simplified coarse-grained representation of each residue (backbone Cα atom plus a pseudoatom representing the side-chain center), retains some sensitivity to side-chain conformations. For models with higher RMSD, the success rate does not continue to decrease and even slightly increase. We examined these cases that found that the higher RMSD is largely due to the relative arrangement of protein subunits in oligomeric complexes, whereas the monomeric units themselves are often predicted accurately. Thus, the increase of the success rate with large RMSD may just imply the accurate sidechain configurations of these oligomeric complexes. Overall, inaccuracies in predicted protein structures can influence success rate to some extent, particularly through side-chain positioning.

The robustness of YuelPocket on the AlphaFold test set is further supported by the high consistency between predictions on experimental (Ground Truth, GT) and AlphaFold (AF) structures (Fig. 4*F*), where ~61% of cases are successful on both, and failures on AF despite success on GT are relatively rare (~13%). A representative example is protein 4I4V (Fig. 4 *G*–*I*). YuelPocket correctly identifies the binding site on both the experimental structure (Fig. 4*G*) and the AlphaFold model (Fig. 4*H*). Notably, subtle side-chain rotations in the predicted structure lead to minor changes in the predicted pocket probability, demonstrating that even though YuelPocket uses a coarse-grained representation (backbone plus side-chain center), it remains sensitive to side-chain conformations. This sensitivity likely contributes to the slight performance decline observed for RMSD values between 0 and 4 Å.

### Comprehensive Binding Site Discovery with Minimal Probe Sets.

**Fig. 5.**
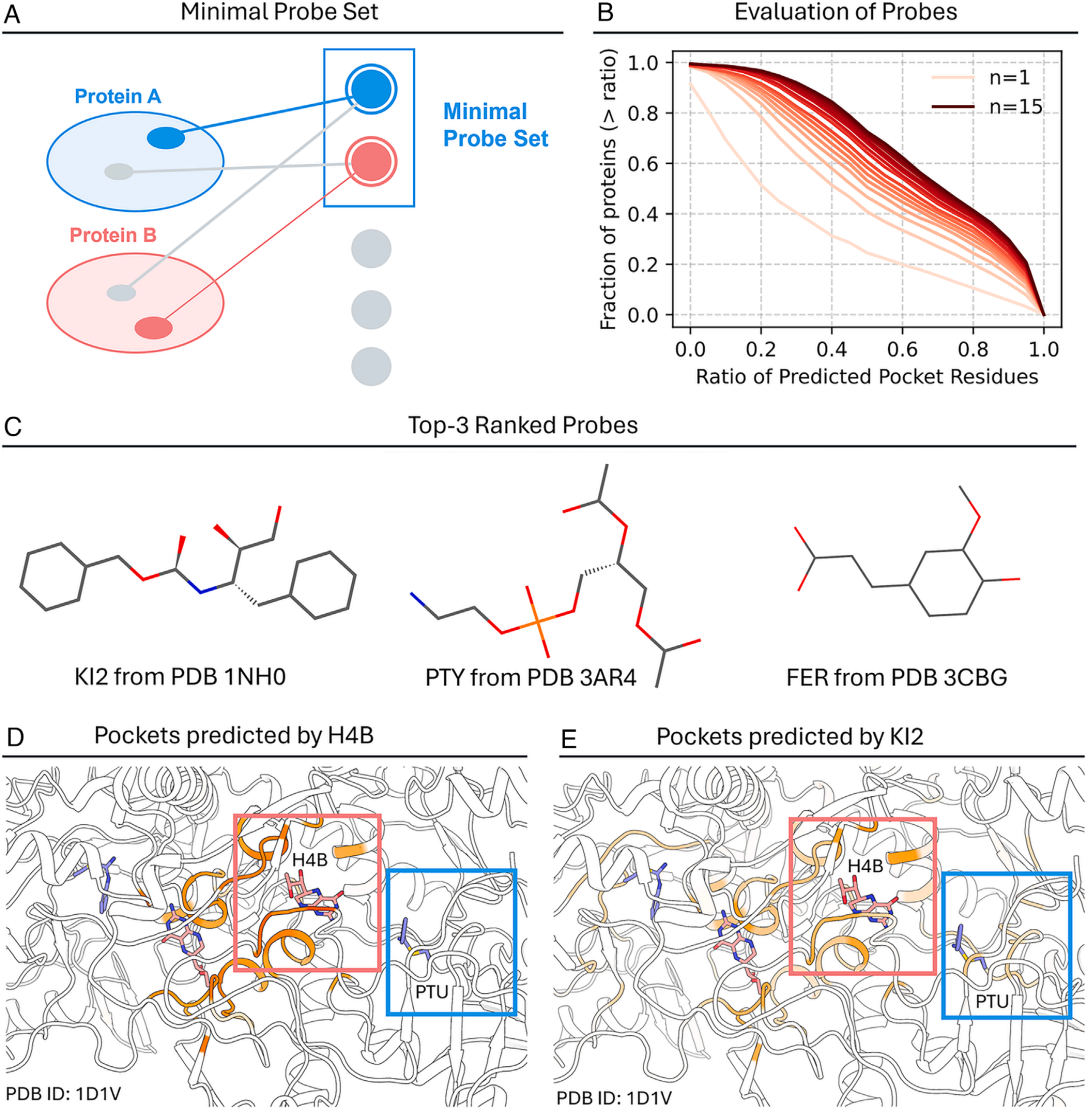
Minimal probe set construction and evaluation for comprehensive binding site mapping. (*A*) Schematic of the definition of minimal probe set. (*B*) Fraction of proteins with the recall of pocket residues greater than the recall threshold vs. the recall threshold. The 15-probe set (red curve) achieves superior coverage compared to single probes. (*C*) Chemical structures of top 3 ranked probes: KI2 (from PDB 1NH0), PTY (from PDB 3AR4), and FER (from PDB 3CBG). (*D*) Native ligand H4B only detects its own binding site, missing the PTU pocket. (*E*) Probe KI2 identifies both H4B and PTU sites, demonstrating pan-pocket coverage.

For some proteins, it is challenging to develop drugs targeting the known active site due to structural constraints, functional requirements, or drug resistance mechanisms. In such cases, identifying allosteric sites (28) or novel binding pockets becomes crucial for drug discovery. Since YuelPocket predicts ligand-specific binding sites, we developed a minimal probe set approach (*Methods* and Fig. 5*A*) to comprehensively identify all potential binding sites across diverse protein targets. A minimal probe set represents an optimized collection of ligands that can collectively cover the majority of binding sites across the entire protein dataset while minimizing redundancy and computational cost.

We implemented a greedy algorithm to construct the minimal probe set, which operates by iteratively selecting ligands that provide the maximum incremental coverage of true pocket residues across the protein dataset. The algorithm begins with an empty probe set and systematically evaluates each candidate ligand based on its ability to identify previously uncovered binding site residues. At each iteration, the ligand that contributes the most new coverage is added to the set, and the process continues until a predefined coverage threshold is achieved. This approach resulted in a minimal probe set of 15 ligands (Top 3 shown in Fig. 5*C*; All shown in *SI Appendix*, Table S1) that provides comprehensive coverage of binding sites across diverse protein families. As the number of probes increases, a larger fraction of proteins achieve recall values above a given threshold for pocket residue identification, demonstrating the effectiveness of the probe set in capturing diverse binding sites (Fig. 5*B*).

Compounds in the minimal probe set usually achieve higher coverage of pocket residues than the other compounds. For example, in protein 1D1V, the known ligand H4B primarily identifies residues around its own binding site, missing the pocket for ligand PTU entirely (Fig. 5*D*). However, when using probe KI2 from our minimal set, the model successfully identifies both the H4B and PTU binding sites, demonstrating the probe’s ability to capture multiple binding pockets within a single protein (Fig. 5*E*). While the prediction probabilities for the H4B site decrease slightly when using KI2, this trade-off is acceptable given the significant gain in overall binding site coverage.

### Correlation between Pocket Probability and Binding Affinity.

We also explored whether the probability of finding a binding pocket for a specific ligand on a protein correlates with the binding affinity between that ligand and protein. This question is particularly significant because YuelPocket was trained exclusively on structural information (protein 3D coordinates and ligand 2D structures) without any exposure to binding affinity data during training. We investigated this relationship using the PDBBind dataset, which contains 5,314 protein–ligand complex structures along with their corresponding Kd and Ki values. For each complex, we computed pocket probabilities using YuelPocket. Since YuelPocket outputs probability scores for individual residues, we employed two aggregation methods to assess correlation with binding affinity: selecting the maximum probability across all residues and calculating the mean probability across all residues.

Our analysis revealed significant correlations between pocket prediction probabilities and binding affinities, despite the model never having been trained on affinity data (*SI Appendix*, Fig. S1). For maximum probability analysis, we observed Pearson correlation of 0.391 and Spearman correlation of 0.423 across all samples (*SI Appendix*, Table S2). Mean probability analysis yielded even stronger correlations, with a Pearson correlation of 0.429 and a Spearman correlation of 0.415 across all samples. These results demonstrate that YuelPocket’s pocket prediction probabilities exhibit moderate to strong correlations with experimental binding affinities, particularly when using mean probability aggregation. This correlation is remarkable because it emerges purely from structural learning without any explicit affinity information during training.

The emergence of binding affinity correlations from purely structural training data has profound implications for our understanding of PLIs. It suggests that the structural features that determine binding site formation are inherently linked to the energetic factors that govern binding strength. This finding further corroborates the fact that the interaction energy must overcome a significant entropic loss due to the binding of a small molecule. Our virtual joint node architecture, by learning to represent the binding pocket as a distinct molecular environment, appears to capture these underlying physical principles that connect structure to function.

## Discussion

A noticeable difference in predictive performance is observed between the Holo4k and PLINDER benchmarks, with consistently higher scores on Holo4k. We attribute this primarily to differences in dataset construction and split strategies. PLINDER was explicitly designed to assess generalization under stringent conditions, enforcing strict separation criteria based on protein sequence similarity, PLI similarity, and ligand chemical similarity. As a result, the PLINDER test split represents a deliberately challenging evaluation set, enriched for binding pockets that are structurally and chemically distinct from those seen during training. Performance on this split therefore reflects a lower-bound estimate of model generalization under strong novelty constraints.

In contrast, although we rigorously filtered Holo4k to remove all samples overlapping with PLINDER training and validation splits, the final filtered Holo4k test set of 340 complexes includes 40 systems shared with the PLINDER test split and 300 systems that do not appear in PLINDER at all. Importantly, PLINDER construction removes a substantial fraction of PDB complexes due to similarity constraints during clustering. The remaining 300 Holo4k-only complexes are therefore likely in the excluded pool and may share higher structural or interaction similarity with training samples than those retained in the PLINDER test split. Consequently, these systems may constitute an intermediate-difficulty regime between in-distribution evaluation and the highly novel PLINDER test set. This difference in effective pocket novelty provides a plausible explanation for the systematically higher performance observed on Holo4k, while remaining fully consistent with the strict generalization behavior measured on PLINDER.

The introduction of the virtual joint node addresses the computational complexity challenge in PLI modeling. An intuitive approach to construct a unified graph is to directly connect all protein residues to all ligand atoms, but it will create a quadratic scaling problem that becomes computationally intractable for large proteins or complex ligands. The edge count in such approach is C + m × n, where *C* represents the summation of the number of protein backbones (Fig. 1*A*), residue contacts (Fig. 1*A*), and compound bonds (Fig. 1*B*), m represents the number of protein residues, and n represents the number of ligand atoms. The m × n term creates quadratic complexity that grows rapidly with molecular size, limiting the applicability of these methods to small proteins and simple ligands. We introduce the virtual joint node as an intermediary that dramatically reduces computational complexity while maintaining information flow between protein and ligand components. In YuelPocket, the edge count is C + m + n, resulting in linear complexity that scales efficiently with molecular size. This reduction enables the model to handle large proteins and complex ligands while maintaining the ability to capture the essential interactions that define binding specificity.

In addition to computational considerations, the accuracy of protein structures can influence residue-level prediction success rates. YuelPocket employs a coarse-grained representation in which each residue is represented by the backbone Cα atom and a pseudoatom corresponding to the side-chain center, similar in spirit to the UNRES (29) force field. This representation is intended to improve model robustness by reducing sensitivity to fine-grained atomic details while retaining essential structural information. Analysis on AlphaFold-predicted structures indicates that small deviations in side-chain conformations, reflected in low RMSD values, can slightly reduce Top-N success rates, suggesting that the model remains partially sensitive to side-chain positioning. Larger RMSD deviations, often arising from intersubunit arrangements in oligomeric proteins, have limited impact on success rates because the pockets of interest may be located within monomeric units. In the future, a simplified representation using only backbone Cα atoms could be explored to further reduce model complexity and enhance generalization. However, such simplification may come at the cost of prediction accuracy, as it would omit information about side-chain geometry that can be critical for identifying precise binding residues. Balancing model robustness and the retention of side-chain information remains an important consideration for improving coordinate- and residue-level prediction performance.

In addition, our results show that the same protein can exhibit dramatically different binding site predictions depending on the input ligand, as demonstrated by the 1DY4 example (*SI Appendix*, Fig. S2), where 1DY4_1 and 1DY4_2 produce distinct binding site patterns. The subtle red coloring observed for 1DY4_1, despite being identical to 1DY4_0, highlights the model’s sensitivity to binding site characteristics: Shallow binding sites with minimal residue–ligand interactions produce lower prediction probabilities, reflecting the reduced binding affinity typically associated with such sites.

## Methods

### Raw Data Collection.

We utilize the PLINDER dataset (v2024-06), a comprehensive and high-quality resource for PLIs, for model training and evaluation. To ensure a rigorous assessment of generalization and minimize information leakage, PLINDER employs a similarity-based clustering strategy for its splits. Specifically, systems are partitioned such that the training, validation, and test sets are separated by thresholds of 30% for protein sequence similarity, 30% for PLI similarity, and 50% for ligand Tanimoto similarity.

Under this schema, the dataset comprises 309,140 systems for training, 832 for validation, and a highly curated test split of 1,036 protein–ligand complexes. The test set is further prioritized for high-quality experimental structures and diversity in pocket types. We trained our model on the full training split and conducted primary evaluations on the 1,036 test systems.

To evaluate the generalization and robustness of the model, two additional test sets were included. The first is Holo4k, an independent benchmark dataset. The original 4,543 systems in Holo4k were rigorously screened against the PLINDER splits, and a subset of 340 complexes was selected to create a challenging test set. This subset includes 40 systems that overlap with the PLINDER test set, ensuring cross-validation consistency, and 300 systems that are entirely absent from PLINDER (training, validation, and test splits), representing novel pockets for strict generalization testing. The second test set consists of AlphaFold-predicted structures corresponding to the 1,036 systems in the PLINDER test set, allowing evaluation of model performance on predicted protein structures rather than experimentally determined ones.

Note that we excluded the widely used COACH420 dataset from this study. Our overlap analysis revealed that the vast majority of COACH420 systems were already present in the PLINDER training data. After filtering for redundancy, the remaining number of unique systems was insufficient to support a statistically meaningful evaluation.

We employ a multistage approach to transform three-dimensional structural information from these datasets into graph representations suitable for deep learning. For each protein–ligand complex, we extract atomic coordinates, residue types, and chemical connectivity patterns through specialized parsing functions.

PDB files are processed to extract residue-level information, where each residue is represented by its Cα atom coordinates and the spatial center of its side chain and one-hot encoded amino acid type $h_{i}\in \{0,1{\}}^{N_{\mathrm{residue}}}$.

### Pocket Detection and GT Generation.

We employ a distance-based approach to identify protein residues that form the binding interface with the ligand. For each protein residue i, we calculate the minimum distance to any ligand atom:

$$d_{min,i}=\min_{j\in \text{ligand}}||x_{protein,i}-x_{ligand,j}|{|}_{2}.$$

A residue is classified as part of the binding pocket if $d_{min,i}\leq \tau_{\mathrm{pocket}}$, where $\tau_{\mathrm{pocket}}=4.0$ Å represents the interaction threshold. This generates binary pocket labels $y\in \{0,1{\}}^{N_{\mathrm{residues}}}$ that serve as GT for supervised learning:

$$y_{i}=\left\{\begin{array}{cc} 1 & \text{if}\;d_{min,i}\leq \tau_{\mathrm{pocket}}\\ 0 & \text{otherwise} \end{array}\right..$$

### Graph Construction.

We represent the protein–ligand system as a unified heterogeneous graph that explicitly models both local atomic interactions and global molecular context. The graph construction process involves three distinct components: the protein subgraph, the ligand subgraph, and a set of virtual “global” nodes that facilitate information exchange.

We parse the protein structure into a dual-node representation for each residue to capture both backbone and side-chain geometry. For each residue, we create 1. A Backbone (BB) node located at the Cα atom, representing the peptide backbone. 2. A Side-Chain (SC) node located at the geometric center of the side-chain atoms, representing the functional part of the residue. Edges are established between protein nodes based on spatial proximity (distance <8.0 Å) to capture the local structural environment. Additionally, we introduce a Protein Virtual Node that connects to all structural nodes (BB and SC) in the protein, serving as a global aggregator of protein information.

Small molecules are represented as molecular graphs where atoms serve as nodes and chemical bonds serve as edges. Similar to the protein, we introduce a Ligand Virtual Node for each ligand molecule that connects to all its constituent atoms. This virtual node aggregates the holistic chemical features of the ligand.

The model processes a batch consisting of the protein graph and multiple ligand graphs, including one true ligand and several negative or decoy ligands. Graph connectivity is defined to capture both local and global interactions: Intraprotein edges represent spatial contacts between protein nodes, intraligand edges correspond to chemical bonds between ligand atoms, and global aggregation edges connect every standard node to its respective virtual node, linking protein nodes to the protein virtual node and ligand atoms to the ligand virtual node.

### Node and Edge Feature Encoding.

Each node $v_{i}$ is initialized with a feature vector $h_{i}\in R^{d_{\mathrm{in}}}$ composed of the one-hot encoding of the amino acid type (for protein nodes) or atom type (for ligand nodes) and the structural mask, which is a 3-dimensional binary mask vector identifying the node type: $\left[mask_{\mathrm{protein}},mask_{\mathrm{ligand}},mask_{\mathrm{virtual}}\right]$.

Edges $e_{\mathrm{ij}}$ carry a 5-dimensional feature vector encoding the interaction type:

$$e_{\mathrm{ij}}=[d_{\mathrm{ij}},I_{\mathrm{contact}},I_{\mathrm{bond}},I_{p\_global},I_{l\_global}],$$

where $d_{\mathrm{ij}}$ is the Euclidean distance (0 for nonspatial edges), and the remaining terms are binary indicators for protein contacts, chemical bonds, protein-to-virtual connections, and ligand-to-virtual connections, respectively.

### Model Architecture.

The YuelPocket architecture is built upon a deep Graph Neural Network (GNN) framework, configured with 16 layers and a hidden dimension of 128. The network processes the unified graph to update node embeddings, allowing information to flow between local geometric neighborhoods and global virtual hubs.

$$H^{\left(l+1\right)},E^{\left(l+1\right)}=\text{GNNLayer}(H^{\left(l\right)},E^{\left(l\right)}).$$

After 16 layers of message passing, the updated node representations are used to perform two distinct but related tasks via specialized prediction heads:

1. Global Pairing Prediction (Contrastive Learning). We explicitly model the compatibility between the protein and the ligand at a global level. This is achieved by interacting the learned representation of the Protein Virtual Node ($h_{P\_Virt}$) with the Ligand Virtual Node ($h_{L\_Virt}$) via an element-wise product, followed by an MLP:

$${\text{Score}}_{\mathrm{pairing}}={\text{MLP}}_{\mathrm{pair}}(h_{P\_Virt}⊙h_{L\_Virt}).$$

During training, we employ a contrastive loss (InfoNCE) to maximize the score of the true protein–ligand pair while minimizing the scores of decoy ligands.

2. Residue-Level Pocket Prediction. To identify the specific binding residues, we compute the interaction between each normal protein node (BB or SC) and the virtual node of the target ligand. For a protein node i with feature $h_{i}$ and the true ligand virtual node with feature $h_{L\_True}$, the pocket probability is computed as

$$P(pocket_{i})=\sigma ({\text{MLP}}_{\mathrm{pocket}}(h_{i}⊙h_{L\_True})).$$

This design allows the model to predict binding sites conditionally based on the specific chemical nature of the input ligand.

### Loss Function.

The model is trained using a multiobjective loss function combining the global pairing objective and the local pocket prediction objective:

$$L_{\mathrm{total}}=L_{\mathrm{pairing}}+L_{\mathrm{pocket}},$$

where $L_{\mathrm{pairing}}$ (Pairing Loss) is the cross-entropy loss identifying the true ligand among 50 random decoys, and $L_{\mathrm{pocket}}$ (Pocket Loss) is a combination of Weighted Binary Cross-Entropy (BCE) to handle class imbalance and Dice Loss to optimize the overlap between predicted and ground-truth pocket regions. The Dice loss is dynamically enabled when the training F1 score exceeds 0.2 to refine the segmentation quality.

### Coordinate-Level Pocket Prediction.

To complement the residue-level predictions, YuelPocket includes a specialized mode designed to directly predict the spatial coordinates of binding pocket centers. This approach reformulates the pocket prediction task as a ranking problem over a set of candidate points generated on the protein surface.

We utilize the Shrake-Rupley algorithm (30) to generate candidate pocket centers on the protein’s SAS. This algorithm samples points on a sphere around each atom (with a radius equal to the atom’s Van der Waals radius plus a probe radius of 1.4 Å) and filters out points that are occluded by neighboring atoms. We treat each surviving SAS point as a potential “probe” or hypothesis for a binding site center. This discretization allows us to comprehensively cover the entire potential binding surface of the protein.

For each candidate probe $p_{k}$, we construct a local interaction graph $G_{k}$ that captures the compatibility between the local protein environment surrounding the probe and the target ligand. The graph nodes include protein residues within a spatial cutoff radius (e.g., 10 Å) of the probe, all atoms of the target ligand, and a single Probe Node representing the candidate center itself. Edges are defined to integrate local and global interactions: Protein–probe edges connect all local protein residues to the Probe Node, ligand-probe edges connect all ligand atoms to the Probe Node—effectively making the probe a “virtual hub” similar to the residue-level model—and internal edges encode standard intraprotein contacts and intraligand bonds. Explicit masks are applied to distinguish protein residues, ligand atoms, and the probe node, ensuring that the model can correctly process the different node types. This construction enables the model to focus on the specific local geometry and physicochemical properties of the protein surface patch defined by the probe.

We train the model using a contrastive learning framework (InfoNCE loss) to distinguish the true binding pocket from various types of decoys. For a given protein–ligand complex, the positive sample is a probe generated near the ground-truth ligand center (distance<4.0 Å). The negative samples (Decoys) are probes generated on the same protein surface but far from the binding site (distance≥4.0 Å) and the true pocket probe paired with a wrong (randomly selected) ligand.

The model scores each probe-ligand pair, and the objective is to maximize the score of the Positive Sample while minimizing the scores of all Decoys. At inference time, we score all SAS probes for a target protein and ligand. The highest-scoring probes represent the predicted binding pocket centers. This approach naturally handles multiple binding sites and provides precise 3D coordinates for downstream applications like docking.

To condense the raw probe scores into discrete binding sites, we employ a Hill-Climbing algorithm combined with Union-Find grouping. Each SAS point “climbs” toward its neighbor with the highest predicted score within its local neighborhood. Points that converge to the same local peak are grouped into the same cluster using the Union-Find data structure. The clusters are then ranked by their peak scores, and the coordinates of the local peaks serve as the predicted pocket centers. This approach naturally handles multiple binding sites and provides precise 3D coordinates for downstream applications like docking.

### Probe Selection Algorithm.

The probe selection algorithm is designed to efficiently identify a minimal set of ligands (probes) that collectively maximize the coverage of true binding pocket residues across a diverse set of protein targets. The process operates in a batch-wise manner, iteratively selecting ligands that contribute the most new coverage of pocket residues for each batch of proteins. For each batch, the algorithm first determines the set of true pocket residues for the proteins in the batch. It then initializes the current coverage using predictions from the already selected probe set. If the current coverage already exceeds a predefined threshold (e.g., 80%), the batch is skipped. Otherwise, the algorithm evaluates each candidate ligand by predicting its pocket coverage across the batch and quantifies how many new, previously uncovered, true pocket residues it identifies. Ligands that contribute new coverage are added to the probe set, and the process continues until the coverage threshold is met or all ligands are exhausted. This greedy, coverage-driven approach ensures that the selected probe set is both efficient and effective in representing the diversity of binding pockets in the dataset.

### Metrics.

To provide a physically meaningful assessment of binding site prediction performance, we prioritize spatial success metrics over standard binary classification metrics such as AUC-ROC or F1-score. Binary metrics are often ill-suited for binding site prediction due to the extreme class imbalance inherent in this task, where the vast majority of residues or spatial points are nonbinding. In addition, these metrics do not account for the spatial proximity between predicted sites and the true ligand location, which is critical for practical applications.

At the residue level, the prediction task is treated as a ranking problem. For a given protein–ligand system, a prediction is defined as a success at Top-K and distance D if at least one of the top K residues, ranked by predicted pocket probability, lies within D Å of any ligand atom. Success rates are reported for Top-1, Top-3, and Top-10 residues across a range of distance thresholds from 4 Å to 10 Å, providing a graded evaluation of how well high-confidence predictions localize binding residues.

For coordinate-level prediction, where discrete pocket centers are identified through clustering of SAS probe points, two standard spatial metrics are used. DCA measures the Euclidean distance between a predicted pocket center and the nearest heavy atom of the ground-truth ligand, while DCC measures the distance between the predicted pocket center and the geometric centroid of the ligand. A prediction is considered successful if the DCA or DCC falls below a specified cutoff, such as 4.0 Å. Success rates are computed as the percentage of test systems in which the Top-1 or Top-3 predicted pocket centers satisfy these criteria. This evaluation framework emphasizes accurate pocket localization, which is directly relevant to downstream tasks such as molecular docking, where precise identification of the binding region is more important than exact boundary delineation.

## Supplementary Material

Appendix 01 (PDF)

## Data Availability

Source codes are deposited at https://github.com/hust220/yuel_pocket (31). Data are deposited at (https://zenodo.org/records/18065818) (32) and (https://zenodo.org/records/16921425) (33).
