# Supplementary material for: Unified protein–small molecule graph neural networks for binding site prediction
Source: Proc Natl Acad Sci U S A. 2026 Mar 3;123(10):e2524913123. doi: 10.1073/pnas.2524913123 (PMC12974528; doi:10.1073/pnas.2524913123)
Supplement: Supplementary file 1 — Appendix 01 (PDF) [file pnas.2524913123.sapp.pdf]

SUPPORTING INFORMATION

Unified Protein-Small Molecule Graph Neural Networks for Binding Site Prediction

Jian Wang[1], and Nikolay V. Dokholyan[1,*]

[1]*Department of Neurology and Neuroscience, University of Virginia, School of Medicine, Charlottesville, VA, United States.*

[*]*Corresponding author: Nikolay V. Dokholyan, E-mail: dokh@virginia.edu.*

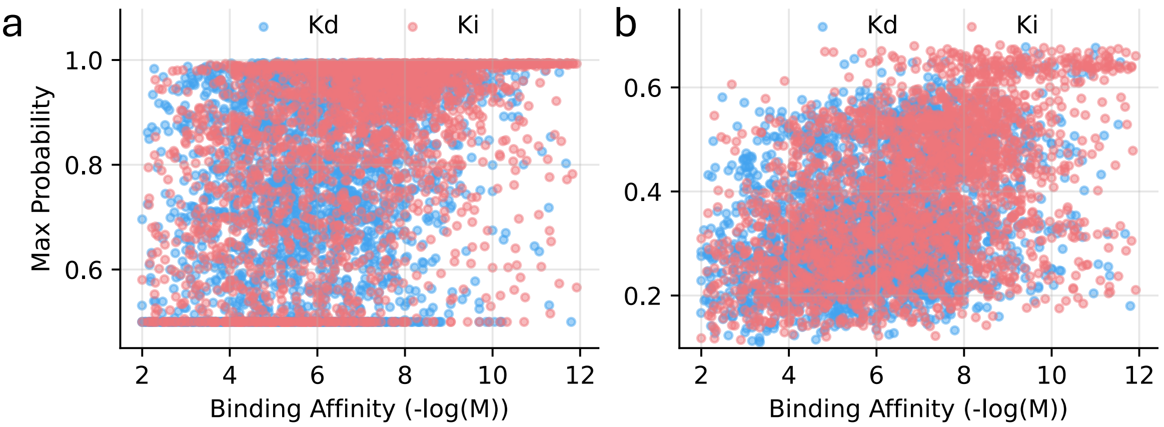

Figure S1. Correlation between pocket probability and binding affinity.

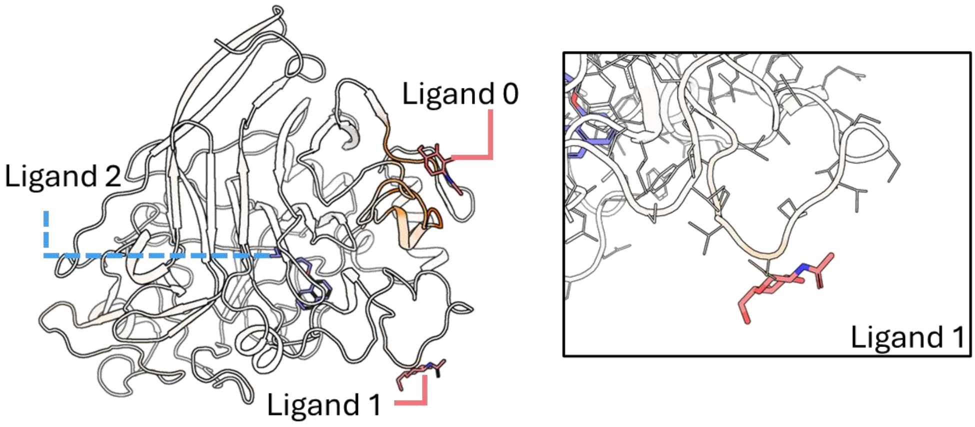

Figure S2. Ligand-specific prediction of 1DY4_1 showing correct identification for ligand 1 but not ligand 2.

Table S1. The 15 probes in the minimal probe set.

PDB_ID	SMILES	size
1nh0_0	OC[C@H](O)[C@H](CC1CCCCC1)N[C@H](O)OCC1CCCCC1	23
1ofz_2	C[C@@H]1O[C@H](O)[C@@H](O)[C@H](O)[C@@H]1O	11
2bxm_0	CCCCCCCCCCCCCC(O)O	16
3ar4_2	CC(O)OC[C@H](CO[PH](O)(O)OCCN)OC(C)O	19

3cbg_1	COC1CC(CCC(O)O)CCC1O	14
3evd_0	NC1NC(O)C2NCN([C@@H]3O[C@H](CO[PH](O)(O)O[PH](O)(O)O[PH](O)(O)O)[C@@H](O)[C@H]3O)C2N1	32
3fp0_1	CC1CC[C@@]2(CO)[C@@H](C1)O[C@@H]1[C@H](OC(C)O)C[C@@]2(C)[C@]12CO2	22
3k5i_2	NC1CNCN1[C@@H]1O[C@H](CO[PH](O)(O)O)[C@@H](O)[C@H]1O	19
3k5i_4	N[C@H]1CNCN1[C@@H]1O[C@H](CO[PH](O)(O)O)[C@@H](O)[C@H]1O	19
3nr4_1	OS(O)(NCC1CCCCN1)C1CCC(Br)C2CCCCC21	22
4csj_0	CC1C[C@@H](C)C[C@H](C)C1S(O)(O)N[C@@H](C)CNC1CCCC2C1CNN2C1CCC(F)CC1	33
4rf9_0	NC(N)NCCC[C@H](N)C(O)O	12
4yha_1	CC(O)NC1S[C@@H](S(N)(O)O)NN1C	14
5tvf_1	NC(NNCC1CCCC(C(N)N)C1)NNCC1CCCC(C(N)N)C1	26
6fu1_0	CCCCCC(O)N(O)[C@H]1C[C@H](O)N(C2CCC(Cl)CC2)C1O	23

Table S2. Correlation between pocket probability and the binding affinity in the PDBBind dataset.

Analysis Method	Affinity Measure	Sample Size	Pearson Correlation	Spearman Correlation
Maximum Probability	All Samples	5314	0.391 (p=1.444e-193)	0.423 (p=1.596e-229)
Maximum Probability	Kd	2783	0.302 (p=1.383e-59)	0.309 (p=1.563e-62)

Maximum Probability	Ki	2531	0.428 (p=4.163e-113)	0.469 (p=1.173e-138)
Mean Probability	All Samples	5314	0.429 (p=4.013e-237)	0.415 (p=8.881e-220)
Mean Probability	Kd	2783	0.306 (p=1.996e-61)	0.290 (p=6.393e-55)
Mean Probability	Ki	2531	0.476 (p=2.310e-143)	0.470 (p=1.340e-139)
